# Conservation analysis of SARS-CoV-2 spike suggests complicated viral adaptation history from bat to human

**DOI:** 10.1093/emph/eoaa041

**Published:** 2020-11-05

**Authors:** Kuan Cheok Lei, Xiaohua Douglas Zhang

**Affiliations:** CRDA, Faculty of Health Sciences, University of Macau, Macau, China

**Keywords:** conservation analysis, SARS-CoV-2, receptor-binding domain, pangolin coronavirus, bat coronavirus RaTG13

## Abstract

**Background:**

The current coronavirus disease 2019 (COVID-19) pandemic, caused by severe acute respiratory syndrome (SARS)-CoV-2, has become the most devastating public health emergency in the 21st century and one of the most influential plagues in history. Studies on the origin of SARS-CoV-2 have generally agreed that the virus probably comes from bat, closely related to a bat CoV named BCoV-RaTG13 taken from horseshoe bat (*Rhinolophus affinis*), with Malayan pangolin (*Manis javanica*) being a plausible intermediate host. However, due to the relatively low number of SARS-CoV-2-related strains available in public domain, the evolutionary history remains unclear.

**Methodology:**

Nine hundred ninety-five coronavirus sequences from NCBI Genbank and GISAID were obtained and multiple sequence alignment was carried out to categorize SARS-CoV-2 related groups. Spike sequences were analyzed using similarity analysis and conservation analyses. Mutation analysis was used to identify variations within receptor-binding domain (RBD) in spike for SARS-CoV-2-related strains.

**Results:**

We identified a family of SARS-CoV-2-related strains, including the closest relatives, bat CoV RaTG13 and pangolin CoV strains. Sequence similarity analysis and conservation analysis on spike sequence identified that N-terminal domain, RBD and S2 subunit display different degrees of conservation with several coronavirus strains. Mutation analysis on contact sites in SARS-CoV-2 RBD reveals that human-susceptibility probably emerges in pangolin.

**Conclusion and implication:**

We conclude that the spike sequence of SARS-CoV-2 is the result of multiple recombination events during its transmission from bat to human, and we propose a framework of evolutionary history that resolve the relationship of BCoV-RaTG13 and pangolin coronaviruses with SARS-CoV-2.

**Lay Summary:**

This study analyses whole-genome and spike sequences of coronavirus from NCBI using phylogenetic and conservation analyses to reconstruct the evolutionary history of severe acute respiratory syndrome (SARS)-CoV-2 and proposes an evolutionary history of spike in the progenitors of SARS-CoV-2 from bat to human through mammal hosts before they recombine into the current form.

## INTRODUCTION

A vigorous outbreak of a novel coronavirus in Wuhan, China, in early 2020 has brought a new wave of concern on the potential risk of coronavirus virulence. Coronaviruses are single-stranded RNA viruses that are capable of imposing health threats to human. Among four genera of coronavirus, namely, alpha-, beta-, gamma and deltacoronavirus, betacoronavirus has the highest medical significance, as it is responsible for not only diseases in those with mild symptoms but also some of the most serious epidemics since the beginning of this century: severe acute respiratory syndrome (SARS) caused by SARS-CoV in 2003, middle-east respiratory syndrome (MERS) caused by MERS-CoV in 2012, and the recent COVID-19 outbreak caused by SARS-CoV-2 in early 2020 [1]. On the other hand, coronaviruses such as human coronavirus (HCoV) OC43 and HKU1, along with alphacoronaviruses HCoV-NL63 and HCoV-229E, cause mild common cold.

The adaptability of coronavirus in mammals and birds gives rise to its diversity and potential virulence to human. Coronaviruses have a wide range of hosts, as suggested by an examination on the deposit in NCBI Genbank that features coronavirus sequences extracted from mammalian animals, such as feline, canine, camels, rodents, swine, cattle, bats, as well as birds. Human coronaviruses have their origins mostly from bats or rodents. Rodent-originated human coronaviruses include HCoV OC43 and HKU1 [2], while others, including HCoV NL63, 229E, SARS-CoV and MERS-CoV, are probably from bat [3]. Bat coronavirus usually passes to human via an intermediate host, as observed in the outbreaks of SARS and MERS in which civet and camel are found to be the respective intermediate hosts [3]. During cross-species transmission, the virus needs to adapt the new host such that it can infect and replicate in the new environment.

There have been numerous studies on the origin and possible transmission pathway of SARS-CoV-2. Bat coronavirus (BCoV) RaTG13 from *Rhinolophus affinis* bat, sharing 96% identity with SARS-CoV-2 genome, is a frequently cited candidate of direct progenitor, while other studies identify pangolin coronavirus (PCoV) as direct progenitor based on the similarity to the receptor-binding domain (RBD) in coronavirus strains in pangolins from wildlife smuggling [4–7]. In general, phylogenetic studies use full-genome sequence, spike and Rdrp (RNA-dependent RNA polymerase) to reconstruct phylogenetic relationship between different CoV strains, especially Rdrp based on its vital importance in replication and thus conservation across species. Nonetheless, the spike gene should also be given more attention, given the crucial role it plays on viral infection.

Viral entry of coronavirus is mediated by a series of molecular interactions between viral surface proteins and host cell receptors. Spike glycoprotein is the major surface protein responsible for host recognition. Spike protein is a type I viral fusion protein, which is primarily characterized by the perpendicular projection of fusion proteins on viral membrane, having an RBD near the N terminus of primary sequence and alpha-helical coiled coil as major secondary structure of fusion subunit [8]. Table 1 summarizes receptor specificities for noted coronaviruses [9, 10]. Mature spike monomers assemble into homotrimers on viral membrane surface that gives the distinctive crown-like structure.

**Table 1.** Human-susceptible coronaviruses and their receptors/targets.

Virus	Genus	Receptor	Optional adhesion factors
SARS-CoV	Betacoronavirus	ACE2	Heparan sulfate proteoglycans, CD209, LSECtin
SARS-CoV-2	Betacoronavirus	ACE2	
MERS-CoV	Betacoronavirus	DDP4 (CD26)	
HCoV-OC43	Betacoronavirus	HLA class I/sialic acids	N-acetyl-9-O-acetylneuraminic acid
HCoV-HKU1	Betacoronavirus	Unknown	O-acetylated sialic acids
HCoV-229E	Alphacoronavirus	APN (CD13)	
HCoV-NL63	Alphacoronavirus	ACE2	Heparan sulfate proteoglycans

ACE2, angiotensin-converting enzyme 2; DDP4, dipeptidyl peptidase 4; APN, aminopeptidase N [9, 10].

Studies have shown that spike RBD itself carries all necessary information for receptor binding in a host-specific manner, as recombinant bat SARSr-CoV Rp3 with replaced RBD gains ability to enter human ACE2-expressing cells [11, 12]. Given the role of spike protein in coronavirus infection, the analysis of the sequences of spike proteins and the characterization of their evolutionary changes help reveal the history of the recent SARS-CoV-2 and identify the source of infectability. In this study, we carried out phylogenetic and sequence analyses to characterize the nucleotide and amino acid sequences of spike proteins in SARS-CoV and SARS-CoV-2 and related CoV strains from public databases, such that we may get insights on the development of human-susceptible coronaviruses from the view of the adaptation of spikes.

## MATERIALS AND METHODS

Viral sequences were downloaded primarily from NCBI Genbank, with pangolin coronavirus genomes obtained from GISAID EpiCoV database by 25 March 2020. The accession numbers of the dataset used can be found in Supplementary Data 1 and 2. Annotated nucleotide sequences of spike were extracted from Genbank feature annotations and were translated into amino acid sequences using BioPython and validated with Genbank record. Multiple sequence alignment was done by MAFFT with default settings except that two guiding trees were built [13]. Phylogenetic tree was constructed using neighbor-joining method, maximum composite likelihood model with Gamma-distributed site rate and 500 bootstrap replications in MEGA version 10.1.7.

Sequence conservation analysis was performed using R package RPHAST 1.6.9 [14]. Nucleotide sequences of spike from 13 CoV strains were selected to generate a comparison of CoV strains between SARS-CoV clade and SARS-CoV-2 clade. Spikes of SARS-CoV-2 were aligned with each of the remaining 12 CoV with MAFFT in setting described above. A neutral model was computed using Jukes-Cantor model (JC69) as a substitution model. The expected length of conserved element was set as 500 and the target coverage as 0.99 to accommodate this dataset. Plots were generated with RPHAST utilities.

Codon adaptation index was calculated according to Sharp and Li [15] in R, using human ribosomal protein as the reference codon set [16]. The relative adaptiveness of the $i^{th}$synonymous codon of the $j^{th}$ amino acid, denoted as $w_{i}$, is defined as

$$w_{i}=\frac{X_{ij}}{\max(X_{j})}\in [0,1]$$

where $X_{ij}$ is the observed count for the $i^{th}$synonymous codon of the $j^{th}$ amino acid and $\max(X_{j})$ is the count of the most abundant codon of the $j^{th}$ amino acid. A minimum of w=0.01 was assigned to codons that were not observed in the reference set. The codon adaptation index (CAI) of a given coding domain sequence (CDS), partitioned as a series of L codons with relative adaptiveness scores $w_{1}$, $w_{2}$, $w_{3}$, …, $w_{L}$, is thus defined as the geometric mean of all scores.

$$\mathrm{CAI}=\sqrt[{L}]{w_{1}\cdot w_{2}\cdot w_{3}\cdot \ldots \cdot w_{L}}={\left(\prod_{i=1}^{L}w_{i}\right)}^{\frac{1}{L}}$$

Codon counts of 1984 human ribosomal protein CDS were obtained from codon usage database of Kazusa Institute (https://www.kazusa.or.jp/codon/). Relative adaptiveness for each codon can be found in Supplementary Data 3. The calculation was done using in-house R script.

Sequence similarity analysis was done with Simplot version 3.5.1 [17]. Spike nucleotide sequence from AY572034 for civet CoV, DQ022305 for BCoV-HKU3, EPI_ISL_410539 for PCoV-17, EPI_ISL_410721 for PCoV-19, AY345986 for SARS-CoV, MN996532 for BCoV-RaTG13, MG772933 for BCoV-ZC45 and MG772934 for BCoVZXC21 were used to compute sequence similarity against MN996527 for SARS-CoV-2, using a window size of 200 bp, a step size of 20 bp and the Kimura distance model. Bootscan analysis employed the same window size and step size, in addition to neighbor-joining tree with 500 bootstrap replications.

Variant analysis of receptor binding domain was done using a Hamming distance-based clustering approach. We referred to currently available studies and identified 24 RBD binding site residues in SARS-CoV-2, which are either involved in direct contact or in proximal distance to ACE2 receptor during the process of host recognition [18–20]. Multiple sequence alignment was then performed on the 13 CoV strains. Homologous residues as well as the corresponding codons in the 12 CoV strains from SARS-CoV-2 clade and SARS-CoV clade were identified by manual inspection. The sum of Hamming distance of codons was calculated for each pair of CoV strains, then hierarchical clustering was performed and visualized with heatmap and principle coordinate analysis.

## RESULTS

Whole-genome phylogenetics reveals a young clade of Sarbecovirus strains.

**Figure 1.**
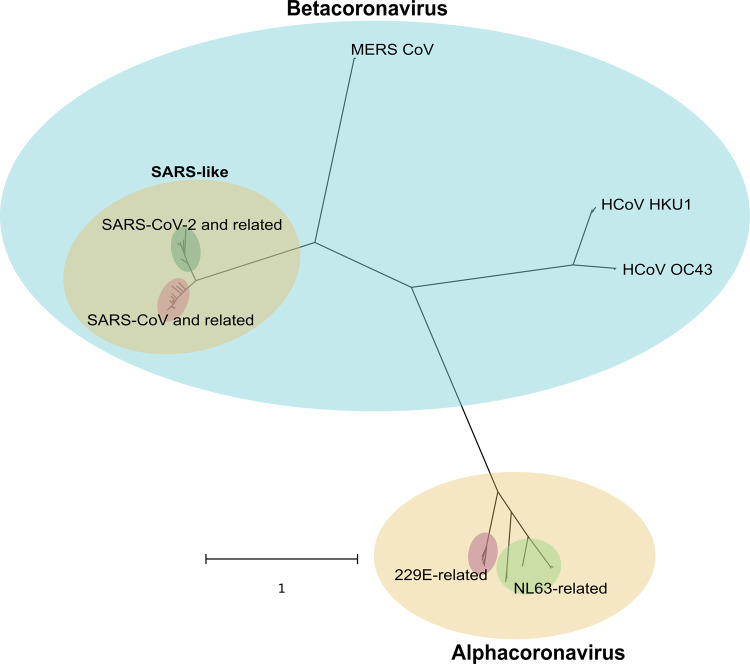
An overview of 995 coronavirus species fetched from NCBI Genbank and GISAID EpiCoV. Detailed taxa are replaced by general grouping

Phylogenetic analysis using the whole genome of 995 coronaviruses categorizes them into alphacoronavirus and betacoronavirus (Fig. 1). HCoV 229E and HCoV NL63 with their respective related viruses share much closer relationship between each other than those in beta-CoV clade, as shown by the close branching points and short branch lengths [21]. In contrast, beta-CoV viruses demonstrates higher variability between each other. From the most recent common ancestor of beta-CoV in this comparison, the first bifurcation produces the last common ancestor (LCA) of lineage A (Embecovirus) and the LCA of lineages B and C. LCA of Embecovirus further evolves and bifurcates into HCoV-HKU1 and HCoV-OC43, respectively [21, 22]. LCA of lineage B-C undergoes another bifurcation event that results in subgenera Merbecovirus (MERS-CoV and related strains) and Sarbecovirus (SARS-like CoV) [22]. MERS-CoV deviates in a greater extent from the LCA of Merbe-Sarbecovirus and yet it presents high similarity within the clade. Finally, SARS-CoV and SARS-CoV-2 with their respective related CoV in animal hosts branch away from their LCA in relatively recent time, as suggested by their proximity to each other at the tip.

**Figure 2.**
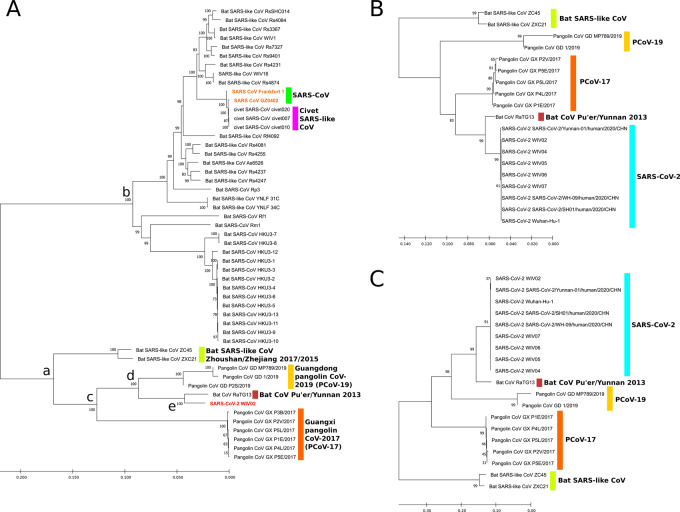
(A) Neighbor-joining phylogenetic tree of SARS-like CoVs from bat and civet, pangolin CoVs, MERS-CoV, SARS-CoV-2 and SARS-CoV using whole-genome sequence. The representation is truncated to remove redundant SARS-CoV and SARS-CoV-2 samples for better visualization. (B) Neighbor-joining phylogenetic trees of amino acid sequences of spike from SARS-CoV-2 clade and SARS-CoV clade. (C) Similar tree to B but constructed using nucleotide sequences

Further inspection on the Sarbecovirus clade shows detailed splitting patterns among closely related CoV. A smaller phylogenetic tree is constructed with only MERS-CoV, SARS-CoV and SARS-CoV-2 with related species, with MERS-CoV set as an outgroup (Fig. 2A, outgroup not shown). From the phylogram two main clades can be identified, representing the SARS-CoV lineage (b) and SARS-CoV-2 lineage (a). In the SARS-CoV-2 group, bat SARS-like CoV strains ZC45 and ZXC21 are probably the earliest to separate from LCA and presumably have the highest similarity to LCA [23]. Two subsequent bifurcation events follow, which gives rise to two groups of pangolin-CoV (PCoV). The earlier one (c) produces PCoV from Guangxi, China, and the later one (d) produces PCoV from Guangdong, China. The final bifurcation (e) yields bat-CoV RaTG13, as agreed by numerous studies to be the closest relative of SARS-CoV-2 [7, 24, 25]. The major branch bifurcations in SARS-CoV-2 clade have branch support values higher than 99%, suggesting a highly confident splitting pattern for the major subclades within SARS-CoV-2-related strains. On the other side, SARS-CoV is related to a larger number of bat SARS-like CoVs in this dataset, with smaller differences to each other suggested by shorter branch lengths. Civet is the most probable intermediate host of SARS-CoV passing from bat to human. Within the SARS-CoV lineage, civet SARS-CoV is nearly identical to SARS-CoV, leaving all bat CoVs as the outgroup. However, in the case of SARS-CoV-2, instead of PCoV being the closest relative, it demonstrates that bat CoV RaTG13 has out-competed all PCoVs and become the closest relative to SARS-CoV-2. Table 2 summarizes the collection dates, submission dates and first publication of coronavirus strains in SARS-CoV-2 clade.

**Table 2.** Information of coronavirus species related to SARS-CoV-2

Accession	Species	Collection date	Submission date	Publication
MG772933	Bat SL-CoV ZC45	Feb 2017	Jan 2018	Hu et al. (2018)
MG772934	Bat SL-CoV ZXC21	Jul 2015	Jan 2018	Hu et al. (2018)
MT072865 EPI_ISL_410543	Pangolin CoV Guangxi/P3B	2017	Feb 2020	Direct submission
MT040333 EPI_ISL_410538	Pangolin CoV Guangxi/P4L	2017	Feb 2020	Direct submission
MT040334 EPI_ISL_410539	Pangolin CoV Guangxi/P1E	2017	Feb 2020	Direct submission
MT040335 EPI_ISL_410540	Pangolin CoV Guangxi/P5L	2017	Feb 2020	Direct submission
MT072864 EPI_ISL_410542	Pangolin CoV Guangxi/P2V	2017	Feb 2020	Direct submission
MT040336 EPI_ISL_410541	Pangolin CoV Guangxi/P5E	2017	Feb 2020	Direct submission
EPI_ISL_410544	Pangolin CoV Guangdong/P2S	2019	Feb 2020	Direct submission
MT121216 EPI_ISL_412860	Pangolin CoV Guangdong/MP789	19 March 2019	Feb 2020	Liu et al. (2020)
EPI_ISL_410721	Pangolin CoV Guangdong/1	2019	Feb 2020	Direct submission
MN996532	Bat CoV RaTG13	24 July 2013	Jan 2020	Zhou et al. (2020)

Direct submission indicates that the sequence is not included in a study when it is submitted to NCBI Genbank.

## SEQUENCE ANALYSIS ON SPIKE

The S gene encodes the spike protein responsible for host recognition, this suggests that its nucleotide and amino acid sequences, implicating its three-dimensional structure, is species-specific and possibly serves as a better candidate in characterizing the adaptation of CoV in different hosts. We extracted nucleotide sequences of the spike from SARS-CoV-2, SARS-CoV, MERS-CoV and bat SARS-like CoV genomes. Since pangolin CoV does not have predefined gene feature annotation, we performed multiple sequence alignment with SARS-CoV-2 and identified the homologous spike regions in pangolin CoV genomes, then translated the identified CDS. We observe a slightly different branching patterns for SARS-CoV-2-related lineage on nucleotide and amino acid sequences. When only spike sequences are considered, in both cases of nucleotide and amino acid, BCoV-RaTG13 has the highest similarity to SARS-CoV-2, a consistent observation with the whole-genome phylogenetic analysis. However, the linking relationships of PCoV-17 and PCoV-19 to SARS-CoV-2 are different between the nucleotide and amino acid trees. For nucleotide sequence, bat-CoV-RaTG13 and SARS-CoV-2 share an LCA, which then shares another LCA with PCoV-19 (Fig. 2C), and this is the same pattern observed in the whole-genome comparison, with branch support value of 98%. On the contrary, in the case of amino acid sequence, the closest PCoV to SARS-CoV-2-BCoV-RaTG13 clade is PCoV-17 (Fig. 2B), though this bifurcation has a much lower branch support value of 83%, which suggests a less robust splitting (<90%). Since its parent branch point and child branch points have >90% branch support values, we still consider the branching between PCoV-17 and SARS-CoV-2-BCoV-RaTG13 clade as credible. In addition, we confirm the insertion of tetrapeptide _681_PRRA_684_ right before the putative S1/S2 cleavage site, as previously reported, to be SARS-CoV-2-exclusive, which is caused by the insertion of _23568_CTCCTCGGCGGG_23579_ that produces an synonymous TCA->TCT mutation of S_680_ followed by _681_PRRA_684_ [26].

## CONSERVATION ANALYSIS ON SPIKE

**Figure 3.**
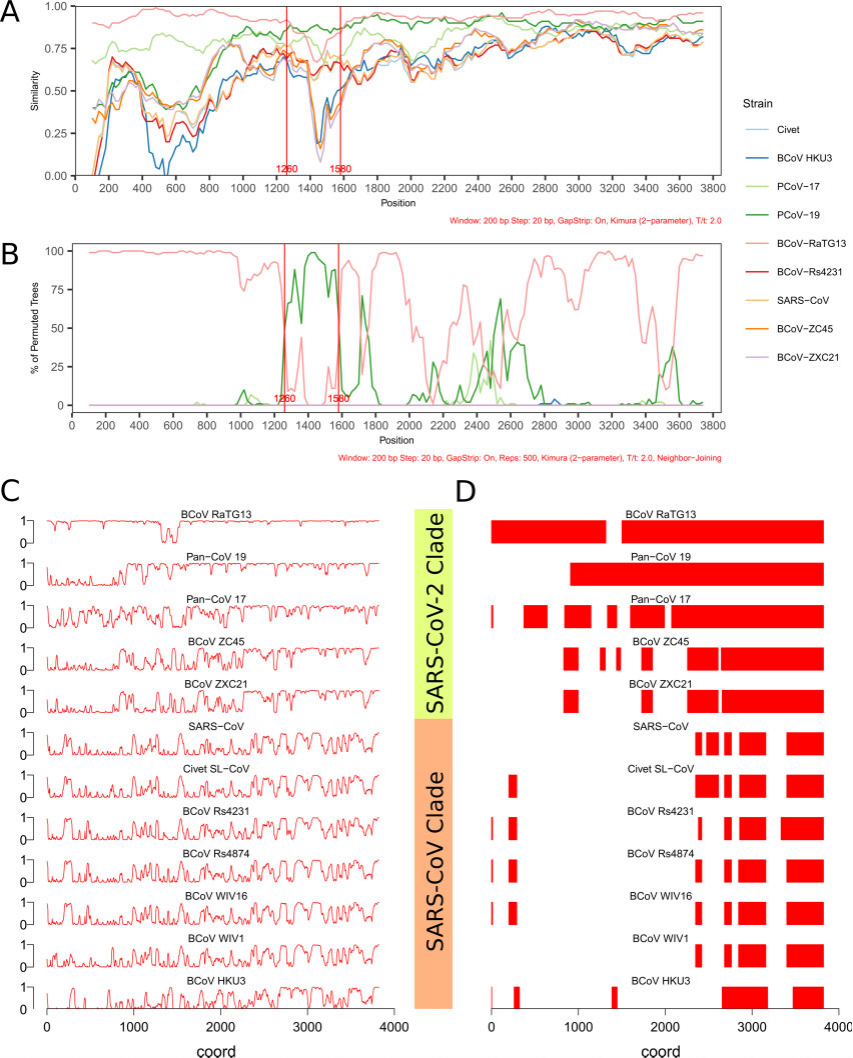
(A) Similarity plot of CoV strains in SARS-CoV-2 clade as well as SARS-CoV and civet SARS-like CoV. Region between 1260 and 1580 bp corresponds to the latter half of RBD, including full RBM. (B) Bootscan plot of CoV strains in SARS-CoV-2 clade as well as SARS-CoV and civet SARS-like CoV. (C) Plot of Phastcons scores along nucleotide sequence of spike in SARS-CoV-2 and SARS-CoV CoV strains. Phastcons score ranges from 0 to 1, the higher the score, the more conserved the region. (D) Plot of conserved elements in the same CoV strains as in (C), showing contiguous regions of high conservation scores in the spike sequence of each strain

Similarity plots of the 12 CoV strains against SARS-CoV-2 spike sequence show that BCoV-RaTG13 has the highest average similarity (92.72%), followed by PCoV-17 (81.56%) and PCoV-19 (80.66%). There is a significant discrepancy between 1260 and 1580 bp, where the average similarity to BCoV-RaTG13 drops to 79.81%, in contrast to the remaining average of 93.96%, while PCoV-19, having 86.50% similarity within this region, becomes dominant (Fig. 3A, Supplementary Data 4) [24, 25]. This suggests a recombination event that alters the composition of amino acid sequence roughly between Y_421_ to P_527_, covering more than half of the RBD and the full extent of receptor binding motif (RBM) [27]. The Bootscan plot provides a clearer representation of possible recombination events (Fig. 3B). The region between 1260 and 1580 bp has the highest probability of being originated from PCoV-19. Similar but weaker signals can also be observed in other loci, such as the peaks around 1720, 2520 and 3580 bp.

In addition, we also observe that BCoV-ZXC21 and BCoV-ZC45, though being classified into the SARS-CoV-2 clade, have strikingly low average similarity in this region (43.13% and 48.38%, respectively), even lower than SARS-CoV and civet-CoV in SARS-CoV clade (67.5% and 67.75%, respectively). Thus, this short sequence may come from an LCA with SARS-CoV in more recent time before it returns to the SARS-CoV-2 clade by homologous recombination.

A more sophisticated conservation analysis was performed to examine these claims. Nucleotide sequences of spike of the 12 CoV strains from SARS-CoV and SARS-CoV-2 clade were aligned with SARS-CoV-2 spike sequence, and conservation scores were calculated and visualized along the alignment to demonstrate the degree of conservation between SARS-CoV-2 and the query sequences. In parallel, conservation scores were also calculated for whole genome alignment (Fig. 3C). The whole-genome conservation analysis demonstrates that the Sarbecovirus species are well conserved in general, especially at the region between 10 000 and 21 000 bp, which corresponds to the end of Orf1a and the full extent of Orf1b in both SARS-CoV-2 clade and SARS-CoV clade species. For SARS-CoV-2 clade, the complete genome sequence is generally conserved except for the S locus, revealing the highly variable nature of spike sequence for host adaptation.

For spike conservation, the results agree with sequence similarity analysis about the close relationship between BCoV-RaTG13 and SARS-CoV-2, with a gap spanning around 1500 bp, which reflects the recombination event mentioned previously. Conserved element plot demonstrates the coherency of the phylogenetic reconstruction of SARS-CoV-2 clade CoV (Fig. 3D). Nucleotide sequence downstream of around 2300 bp, corresponding to the S2 subunit, is largely conserved among SARS-CoV-2 clade species. The extent of conservation in S1 subunit is then observed to be correlated to the phylogenetic relationship. It is worthy to mention that the first 1000 bp of SARS-CoV-2 spike sequence, corresponding to the N-terminal domain (NTD) region, has low conservation scores when compared with PCoV-19, but relatively higher with PCoV-17. This observation can be reaffirmed by sequence similarity analysis, in which PCoV-17 rather than PCoV-19 has higher similarity next to BCoV-RaTG13 in the NTD (76.32% vs 51.10%). Since it is not evident that the spike NTD has undergone homologous recombination with PCoV-17, we propose that the similarity is a result of cumulative mutations in the course of natural selection. In summary, there is a higher probability that SARS-CoV-2 and BCoV-RaTG13 share the most recent LCA than any other CoV strains in SARS-CoV-2 clade in our current study. The NTD of spike may have evolved separately from the latter part of the gene body, then recombined with the LCA of BCoV-RaTG13 and SARS-CoV-2.

## CODON ADAPTATION IN SPIKE

CAI is a widely used metric to infer the overall adaptiveness of a coding sequence given a reference set of codon usage [15]. The CAI of a coding sequence is defined as the geometric mean of relative adaptiveness of codons, which is in turns defined as the frequency of that codon divided by the frequency of the most abundant codon of the same amino acid in a reference set. The reference codon usage is usually derived from perpetually translated proteins, which reflects the optimal tRNA abundance of the species. As viral translation depends on host cell machineries, human ribosomal proteins are used as the reference.

**Figure 4.**
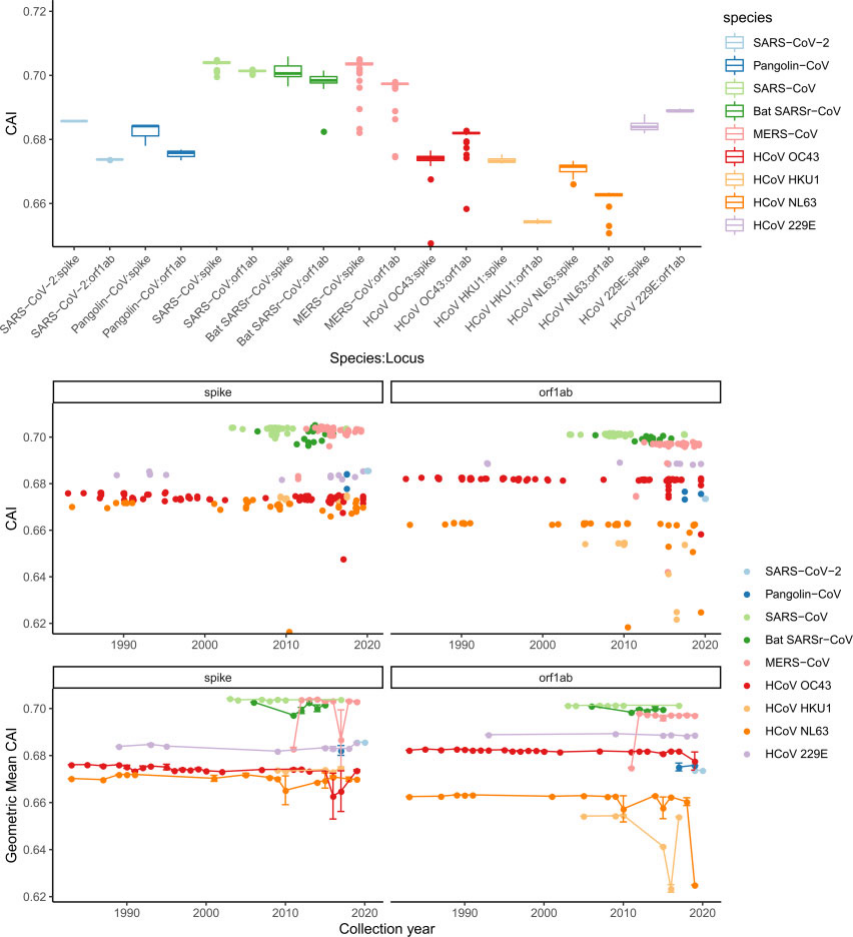
Upper: Codon adaption index calculated using spike and Orf1ab CDS regions in different groups. Middle: CAI for each sample is calculated and is arranged according to collection time, plots are created to demonstrate the difference between CAI for spike and CAI for orf1ab. Lower: Like the middle plot, but CAI for each year is averaged as geometric mean

A comparison of CAI of spikes of SARS-CoV-2, SARS-CoV, pangolin CoV and bat SARSr-CoV infers the similarity of codon usage bias to the reference of the protein in a host cell. Figure 4 shows a boxplot (upper) of the CAI values of CDS of spike and orf1ab in different CoV strains. The general trend is that the spike has a larger CAI value than orf1ab, except for HCoV OC43 in which the CAI of orf1ab is larger than that of the spike. SARS-CoV, bat SARSr CoV and MERS-CoV have higher CAI values within the same range (0.696–0.704), while SARS-CoV-2 and pangolin CoV sit in a lower CAI range along with HCoV OC43 (0.674–0.686).

For the spike CDS, SARS-CoV-2 (mean CAI = 0.686) and pangolin CoV (0.682, 0.6801–0.6843) can be classified into one group, while SARS-CoV (mean CAI = 0.704, 0.7036–0.7038) and bat SARSr-CoV (0.701, 0.7007–0.7015) go into another group with higher CAI values. Similar pattern is observed for the Orf1ab CDS, which encodes non-structural viral enzymes and thus is assumed to be more prone to host codon optimization. The Orf1ab in SARS-CoV-2 (0.674) and pangolin-CoV (0.675, 0.6744–0.6764) still has smaller CAI values than in SARS-CoV (0.701) and bat SARSr-CoV (0.698, 0.6976–0.6986). This difference can be visualized better by plotting CAI against collection time. For the spike, the CAI scores of SARS-CoV, bat SARSr-CoV and MERS-CoV are the highest among all and occupy the top region in the plot, while the CAI scores of the four HCoV species occupy a lower region (Fig. 4, middle and lower). The CAI scores of SARS-CoV-2 and pangolin CoV strains sit between these two groups, closer to HCoV and overlapping with HCoV 229E. For the CAI scores of orf1ab, a similar pattern for SARS-CoV and MERS-CoV can be observed. One important feature of these plots of CAI scores against time is that the scores remain constant regardless of the type of CDS (non-structural/structural) and species. Considering that all CAI scores were calculated using human reference, it suggests that for either SARS-CoV-2 or SARS-CoV and their related strains, a change in the host does not affect the range of CAI. Instead, we observe that the coronavirus strains with the highest CAI scores are those resulting in higher mortality rate. The CoV strains causing common cold have lower CAI scores, SARS-CoV and MERS-CoV have higher, and SARS-CoV-2 sits in the middle of them.

In summary, this result suggests that SARS-CoV-2 has lower usage of the most abundant codons in human than SARS-CoV. The CAI scores do not appear to increase or decrease significantly through time, implying that the optimization of codon usage in a new host is not a main driving force of the evolution of the coronaviruses, and also not a significant factor for the prevalence of the coronaviruses. The result implies that CAI scores are more correlated to the phylogenetic grouping of the coronaviruses (Table 3). 

**Table 3.** The number of sequences being assessed (N), mean, standard deviation and standard error of codon adaptation index in 7 CoV strains for spike and orf1ab coding domain sequences.

Species	N	Mean	Standard deviation	Standard error
Spike				
SARS-CoV-2	7	0.6857	0.0000	0.0000
Pangolin-CoV	3	0.6822	0.0036	0.0021
SARS-CoV	186	0.7037	0.0009	0.0001
Bat SARSr-CoV	35	0.7011	0.0025	0.0004
MERS-CoV	338	0.7023	0.0157	0.0009
HCoV OC43	170	0.6729	0.0137	0.0010
HCoV HKU1	38	0.6734	0.0007	0.0001
HCoV NL63	54	0.6699	0.0075	0.0010
HCoV 229E	15	0.6842	0.0018	0.0005
Orf1ab				
SARS-CoV-2	7	0.6737	0.0000	0.0000
Pangolin-CoV	3	0.6754	0.0017	0.0010
SARS-CoV	174	0.7013	0.0003	0.0000
Bat SARSr-CoV	33	0.6981	0.0031	0.0005
MERS-CoV	326	0.6963	0.0133	0.0007
HCoV OC43	151	0.6817	0.0021	0.0002
HCoV HKU1	37	0.6523	0.0073	0.0012
HCoV NL63	54	0.6608	0.0080	0.0011
HCoV 229E	14	0.6889	0.0004	0.0001

## DISTANCE ANALYSIS ON RBD BINDING SITES

**Figure 5.**
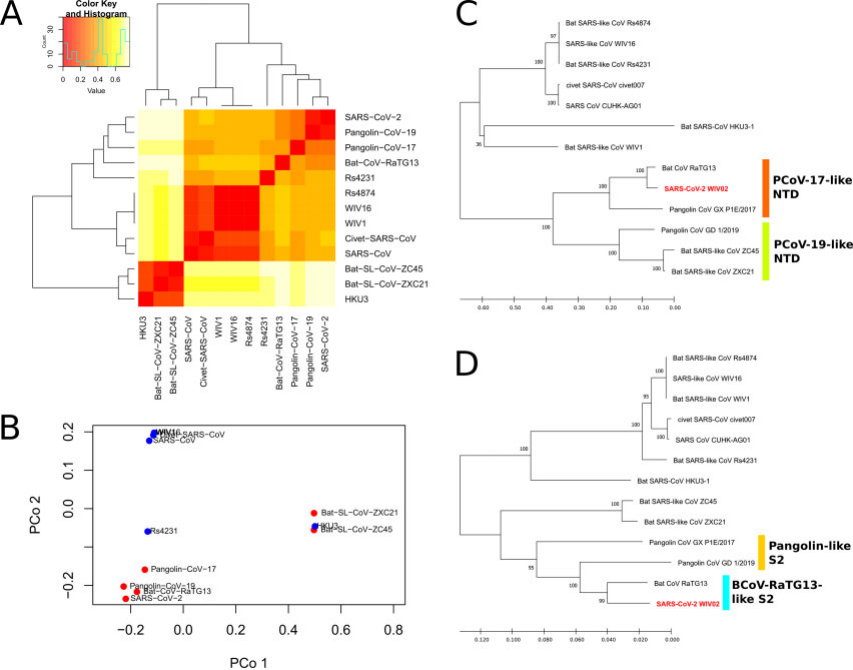
(A) Heatmap visualization and clustering of the 13 CoV strains based on the sum of Hamming distance of codons corresponding to 24 RBD binding sites. Three clusters can be observed: one cluster for SARS-CoV-2, pangolin CoV and related strains, one for SARS-CoV and related strains, and the last for BCoV-ZC45, BCoV-ZXC21 and BCoV-HKU3. (B) PCoA visualization of Hamming distance of the 13 CoV strains. (C) Neighbor-joining tree of the 13 CoV strains using the first 900 bp of spike nucleotide sequence, corresponding to the NTD region. (D) Neighbor-joining tree of the 13 CoV strains using the S2 subunit region of spike

Twenty-four sites on the RBD of SARS-CoV-2 are identified from literature and they are compared across closely related CoV as well as SARS-CoV and SARS-like CoV (Tables 4 and 5) [18–20]. We observe that only 8 out of 24 residues (33.3%) are identical between SARS-CoV and SARS-CoV-2, which is lower than that in bat-CoV-RaTG13 (15, 62.5%), PCoV-17 (16, 66.7%) and PCoV-19 (22, 91.7%). On the other hand, SARS-CoV and civet SARS-CoV have 20 (83.3%) identical residues, which is the same as some other CoVs, such as bat-SL-CoV-Rs4874 and WIV16 and WIV1 (20, 83.3%), and is distant from the remaining related CoVs like bat-SL-CoV-Rs4231 (11, 45.8%) and HKU3 (5, 20.8%). Considering the codons encoding the residues, we use the codons at each site in each species to compute a distance matrix, in which the distance represents the Hamming distance of all codons in one species to all codons in another species (Fig. 5A). Three clusters can be observed in the heatmap, one for SARS-CoV-2 and pangolin-CoV-2019, one for SARS-CoV, civet SARS-CoV, bat SARS-like CoVs WIV1, WIV16 and Rs4874, and the remaining for bat SARS-like-CoVs ZC45, ZXC21 and HKU3. It suggests that considering only receptor binding sites in RBD, pangolin-CoV-19 has the largest possibility to be able to recognize human ACE2 as well as SARS-CoV-2. As previously mentioned, SARS-CoV-2 has largely conserved spike sequence with BCoV-RaTG13 except for RBD, which probably comes from PCoV-19.

**Table 4.** Table of residues at the 24 RBD binding sites in SARS-CoV-2 and corresponding residues in the 12 CoV strains, crucial binding sites are set in italics

Pos	SARS- CoV-2	Bat- CoV- RaTG13	Pangolin- CoV-17	Pangolin- CoV-19	Bat-SL- CoV	SARS- CoV	Civet- SARS- CoV	Rs4231	WIV16	Rs4874	WIV1	HKU3
417	K	K	V	R	V	V	V	V	V	V	V	V
439	N	K	V	N	A	R	R	N	R	R	R	A
445	V	E	T	V	–	S	S	T	Q	Q	Q	–
446	G	G	G	G	–	T	T	S	T	T	T	–
449	Y	F	Y	Y	–	Y	Y	Y	Y	Y	Y	–
453	Y	Y	Y	Y	Y	Y	Y	Y	Y	Y	Y	Y
*455*	*L*	*L*	*L*	*L*	*S*	*Y*	*Y*	*W*	*S*	*S*	*S*	*S*
456	F	F	F	F	H	L	L	V	L	L	L	H
473	Y	Y	Y	Y	–	F	F	Y	F	F	F	–
474	Q	Q	Q	Q	–	S	S	S	S	S	S	–
475	A	A	A	A	–	P	S	P	P	P	P	–
484	E	T	V	E	R	A	A	G	A	A	A	Y
485	G	G	G	G	–	–	–	–	–	–	–	–
*486*	*F*	*L*	*L*	*F*	*-*	*L*	*P*	*P*	*F*	*F*	*F*	*G*
487	N	N	N	N	N	N	N	N	N	N	N	N
489	Y	Y	Y	Y	V	Y	Y	Y	Y	Y	Y	V
*493*	*Q*	*Y*	*E*	*Q*	*S*	*N*	*R*	*R*	*N*	*N*	*N*	*S*
496	G	G	G	G	D	G	G	G	G	G	G	D
498	Q	Y	H	H	N	Y	Y	F	Y	Y	Y	N
500	T	T	T	T	N	T	T	T	T	T	T	N
*501*	*N*	*D*	*T*	*N*	*V*	*T*	*S*	*A*	*N*	*N*	*N*	*V*
502	G	G	G	G	P	G	G	G	G	G	G	P
503	V	V	V	V	L	I	I	V	I	I	I	V
*505*	*Y*	*H*	*Y*	*Y*	*Y*	*Y*	*Y*	*H*	*Y*	*Y*	*Y*	*Y*

**Table 5.** Table of codons for residues at 24 RBD binding sites in SARS-CoV-2 and corresponding residues in 12 CoV strains

Pos	SARS- CoV-2	Bat-CoV- RaTG13	Pangolin- CoV-17	Pangolin- CoV-19	Bat-SL-CoV- ZXC21	Bat-SL- CoV-ZC45	SARS-CoV	Civet- SARS-CoV	Rs4231	WIV16	Rs4874	WIV1	HKU3
1249	AAG	AAG	GTT	AGA	GTT	GTC	GTT	GTT	GTT	GTT	GTT	GTT	GTT
1315	AAC	AAG	GTT	AAC	GCA	GCC	AGG	AGG	AAT	AGG	AGG	AGG	GCT
1333	GTT	GAG	ACT	GTT	AAA	AAA	TCA	TCA	ACT	CAA	CAA	CAA	AAA
1336	GGT	GGC	GGT	GGT	–	–	ACT	ACT	TCC	ACT	ACT	ACT	–
1345	TAT	TTT	TAT	TAT	–	–	TAT	TAT	TAT	TAT	TAT	TAT	–
1357	TAT	TAC	TAT	TAT	TAT	TAC	TAT	TAT	TAT	TAT	TAT	TAT	TAC
1363	TTG	CTC	TTA	TTG	TCT	TCT	TAT	TAT	TGG	TCT	TCT	TCT	TCT
1366	TTT	TTT	TTT	TTT	CAT	CAT	CTT	CTT	GTT	CTC	CTC	CTC	CAT
1417	TAT	TAC	TAC	TAC	–	–	TTC	TTC	TAT	TTC	TTC	TTC	–
1420	CAG	CAA	CAA	CAA	–	–	TCC	TCT	TCA	TCT	TCT	TCT	–
1423	GCC	GCA	GCC	GCT	–	–	CCT	TCT	CCT	CCT	CCT	CCT	–
1450	GAA	ACT	GTT	GAA	–	–	CCT	CCT	ATA	CCT	CCT	CCT	–
1453	GGT	GGT	GGT	GGT	–	–	GCT	GCT	GGT	GCT	GCT	GCT	–
1456	TTT	CTA	CTA	TTT	GAG	GAG	CTT	CCT	CCT	TTT	TTT	TTT	GGT
1459	AAT	AAT	AAT	AAC	AAT	AAT	AAT	AAT	AAT	AAT	AAT	AAT	AAT
1465	TAC	TAC	TAT	TAC	GTC	GTC	TAT	TAT	TAT	TAT	TAT	TAT	GTG
1477	CAA	TAT	GAA	CAA	AGT	AGT	AAT	AGA	CGT	AAT	AAT	AAT	TCA
1486	GGT	GGA	GGT	GGT	GAC	GAC	GGT	GGT	GGC	GGT	GGT	GGT	GAC
1492	CAA	TAC	CAC	CAC	AAC	AAC	TAC	TAC	TTT	TAC	TAC	TAC	AAC
1498	ACT	ACT	ACT	ACT	AAT	AAT	ACT	ACT	ACA	ACT	ACT	ACT	AAC
1501	AAT	GAT	ACA	AAT	GTA	GTA	ACT	AGT	GCT	AAT	AAT	AAT	GTT
1504	GGT	GGT	GGT	GGT	CCG	CCA	GGC	GGC	GGT	GGC	GGC	GGC	CCA
1507	GTT	GTT	GTT	GTT	CTT	CTT	ATT	ATT	GTT	ATA	ATA	ATA	GTA
1513	TAC	CAC	TAC	TAC	TAT	TAC	TAC	TAC	CAC	TAC	TAC	TAC	TAT

Moreover, bat-SL-ZC45 and ZXC21, which are always grouped together with SARS-CoV-2 in contiguous spike sequences, are now displaying a different clustering pattern, that they cluster with BCoV-HKU3 in SARS-CoV clade. In reference to the hierarchical clustering in Fig. 5A, we propose that BCoV ZXC21, ZC45 and HKU3 may be related to the early variant of human-insusceptible bat CoV derived from the LCA of all Sarbecovirus. Since SARS-CoV and SARS-CoV-2, both recognizing human ACE2, are clustered into a larger subgroup while having distant relationship within the subgroup, it implies that the human-susceptibility in the two CoVs evolves independently, thus having a larger edit distance with each other. Nonetheless, it may be speculated that any of the CoV strains within the subgroup will be capable of evolving into human-susceptible RBD binding sites.

## DISCUSSION

We have shown with phylogenetic analyses different clustering patterns of SARS-CoV-2-related species using either whole genome or spike sequences. The relationship between the CoV strains in SARS-CoV-2 clade is a disputable matter. Jaimes et al. [26] produced a tree using SARS-CoV-2 spike protein sequences, it agrees with the bifurcation order of BCoV ZC45, ZXC21, and RaTG13 with SARS-CoV-2 but they did not include pangolin CoV in their tree. One study used the concatenated genomic sequence of ORF1ab, S, E, M and N genes to produce a tree, which included all pangolin CoVs used in this study, and it gives the same branching pattern in SARS-CoV-2 clade as our tree using unedited whole-genome sequence [28]. Moreover, Lam et al. compared the phylogenetic trees using two regions (Regions 4 and 5) covering the end of ORF1ab and a portion of S1 subunit of the spike. Both trees agree that BCoV-RaTG13 has the highest similarity to SARS-CoV-2 in the compared regions. However, the tree for region 5, corresponding to the RBD region, demonstrated that bat CoV ZXC21 and ZC45 group with bat SARS CoV HKU3 and Rf1, both belonging to SARS-CoV-related clade as shown in this study. This clustering pattern can also be observed in our mutation analysis of RBD, we thus reaffirm that this region is highly sensitive to host adaptation and that the RBD in BCoV-ZXC21 and ZC45 is highly similar to the early form of Sarbecovirus RBD.

Our mutation analysis on the RBD region focuses exclusively on the contact sites of SARS-CoV-2 spike protein with the human ACE2 protein. The result demonstrates that pangolin CoV, rather than bat CoV RaTG13, has the highest similarity to SARS-CoV-2. In comparison, Lam et al. [28] have also produced two similar trees with SARS-CoV-2 related CoVs using nucleotide sequences of RBD with lengths at around 570 bases (i.e. 190 residues), one tree for full sequence and one tree for synonymous sites only. Neither tree has the exact branching as our tree, but it also demonstrated that SARS-CoV-2 and PCoV-19 has the highest similarity when compared using all bases in the RBD sequence. The authors suggested that the similarity of RBD between pangolin CoV and SARS-CoV-2 is caused by convergent evolution rather than recombination. The tree with synonymous sites represents a neutral selection model without recombination, and it shows that the spike of SARS-CoV-2 has the closest relationship to the one in bat CoV RaTG13. Nonetheless, the result of our RBD analysis demonstrates that the evolution of spike to become human-susceptible probably occurs inside an intermediate pangolin host.

**Figure 6.**
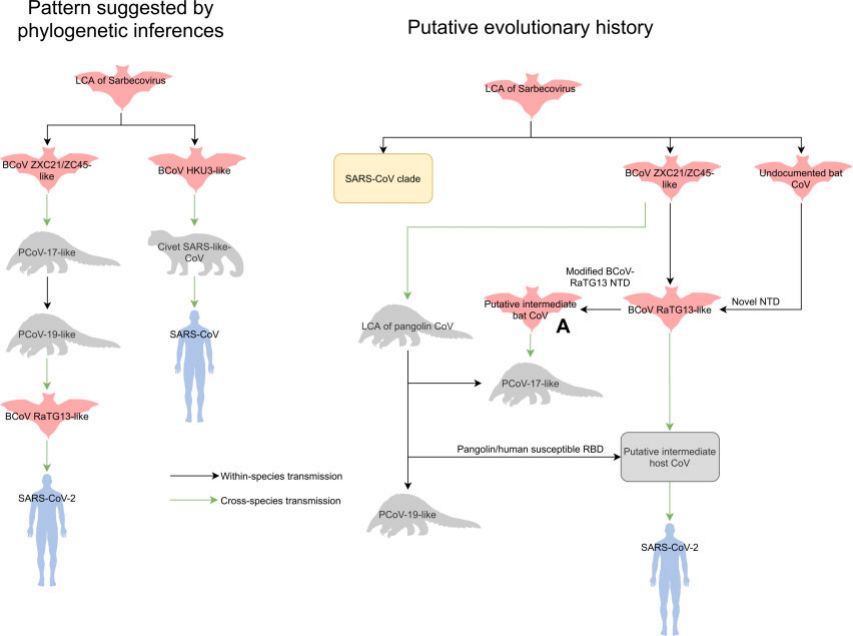
Schematic diagram of possible evolutionary history. Left: Pattern suggested based on full-genome phylogenetic inference. Right: Putative order based on analyses of spike sequences, introducing possible intermediates to explain the discrepancies in phylogenetics of NTD, RBD and S2 subunit of spike nucleotide sequence

The phylogenetics of both whole genome and spike sequences suggest an apparently direct passage of SARS-CoV-2 from bat to human, but the distance analysis on RBD rejects this pattern. Based on the analysis of spike sequences, it is reasonable to believe that pangolins or related mammals are the intermediate host, but the transmission takes more complicated steps. The existence of a conserved element in the NTD in BCoV-RaTG13 and SARS-CoV-2 but not elsewhere implies independent lineages of pangolin CoV and SARS-CoV-2/BCoV-RaTG13 since early time. The pangolin lineage may be originated from BCoV-ZXC21/ZC45 via cross-species transmission and it becomes a sister lineage of SARS-CoV-2/BCoV-RaTG13. The LCA of pangolin CoV may have bat-like RBD, but subsequently a pangolin-specific RBD will be developed. The NTD in BCoV-RaTG13 may have resulted from a recombination with an undocumented bat Sarbecoviruses such that it does not cluster with BCoV-ZXC21/ZC45. PCoV-17 may have emerged earlier than PCoV-19, then undergone a recombination to obtain NTD from BCoV-RaTG13 lineage through an intermediate bat CoV (A, Fig. 6) with modified NTD sequences. This would explain the similarity in NTD between BCoV-RaTG13, PCoV-17 and SARS-CoV-2, while keeping PCoV-17 in pangolin lineage. In the final stage, BCoV-RaTG13, which provides most of the spike sequence except the RBD, must recombine with the RBD of PCoV-19. The phylogenetics of NTD, RBD and S2 subunit imply that the recombination only replaces RBD from BCoV-RaTG13 spike sequence. The final intermediate host is not necessarily pangolins, but they would be susceptible to both the infection of BCoV-RaTG13 and PCoV-19.

This hypothesis assumes that (i) CoV transmits from bats to intermediate mammals, and then to human in a single-way fashion, i.e. without backward transmission and (ii) cross-species transmission does not significantly alter the spike sequence except for the RBD region, and any substantial changes in sequence other than RBD region are the result of recombination. This hypothesis predicts the existence of a bat SARS-like CoV of which the NTD of spike will cluster with BCoV-RaTG13 better than with SARS-CoV-2, while in whole-genome phylogenetics it will be the sister species of BCoV-ZXC21/ZC45. Further investigation and analyses will be required to test the hypothesis. 

## Supplementary Material

eoaa041_Supplementary_Data
